# Unexpected decrease of full-length prion protein in macaques inoculated with prion-contaminated blood products

**DOI:** 10.3389/fmolb.2023.1164779

**Published:** 2023-05-05

**Authors:** Nina Jaffré, Jérôme Delmotte, Jacqueline Mikol, Jean-Philippe Deslys, Emmanuel Comoy

**Affiliations:** Commissariat à l’Energie Atomique, DRF/IBFJ/SEPIA, Fontenay-aux-Roses, France

**Keywords:** prion, prion disease, v-CJD, non-human primate, myelopathy, blood transfusion, PrP, C1 fraction

## Abstract

The presence of prion infectivity in the blood of patients affected by variant Creutzfeldt–Jakob disease (v-CJD), the human prion disease linked to the bovine spongiform encephalopathy (BSE), poses the risk of inter-human transmission of this fatal prion disease through transfusion. In the frame of various experiments, we have previously described that several cynomolgus macaques experimentally exposed to prion-contaminated blood products developed c-BSE/v-CJD, but the vast majority of them developed an unexpected, fatal disease phenotype focused on spinal cord involvement, which does not fulfill the classical diagnostic criteria of v-CJD. Here, we show that extensive analyses with current conventional techniques failed to detect any accumulation of abnormal prion protein (PrP^v−CJD^) in the CNS of these myelopathic animals, i.e., the biomarker considered responsible for neuronal death and subsequent clinical signs in prion diseases. Conversely, in the spinal cord of these myelopathic primates, we observed an alteration of their physiological cellular PrP pattern: PrP was not detectable under its full-length classical expression but mainly under its physiological terminal-truncated C1 fragment. This observed disappearance of the N-terminal fragment of cellular PrP at the level of the lesions may provide the first experimental evidence of a link between loss of function of the cellular prion protein and disease onset. This original prion-induced myelopathic syndrome suggests an unexpected wide extension in the field of prion diseases that is so far limited to pathologies associated with abnormal changes of the cellular PrP to highly structured conformations.

## Introduction

The cellular prion protein (PrP^c^) is a highly conserved glycoprotein expressed in many different tissues, with the highest levels found in the central nervous system (CNS). This protein (231 amino acids in humans) is an extracellular α-helix-rich protein topped with a flexible unstructured N-terminal domain (amino acids 23–120) and is anchored to the cell membrane by a glycophosphatidylinositol (GPI) anchor. Its conversion into a partly protease-resistant, β-sheet-rich misfolded isoform, called PrP^d^ under a generic term (disease-linked prion protein), and its accumulation in aggregative forms in the CNS are hallmarks of fatal neurodegenerative disorders that are known for decades as transmissible spongiform encephalopathies, or prion diseases (Gajdusek, 1977; Prusiner, 1991). They affect humans with Creutzfeldt–Jakob disease, Gerstmann–Sträussler–Scheinker syndrome (GSS), fatal familial insomnia (FFI), and Kuru, and they affect animals with scrapie in small ruminants, bovine spongiform encephalopathy (BSE) in cattle, and chronic wasting disease (CWD) in cervids.

The physiological functions of PrP^c^ are still under discussion (Kovač and Čurin Šerbec, 2022). Several experiments indicate that PrP^c^ is involved in physiological processes, including neurogenesis, neuroprotection, copper homeostasis, synaptic plasticity, myelin sheath maintenance, and cellular signaling pathways (Mitteregger et al., 2007; Bremer et al., 2010; Altmeppen et al., 2012) and also in pathological mechanisms of neurodegenerative diseases such as Alzheimer’s and Parkinson’s diseases (Brody and Strittmatter, 2018; Nieznanska et al., 2021; Shafiq et al., 2022). PrP^c^ is subject to proteolytic processing under physiological conditions, mainly by alpha-cleavage liberating N1 and C1 (cleavage in the human sequence between amino acids 111–112), considered to be the biologically active fragments of the PrP^c^ (Altmeppen et al., 2013). A beta-cleavage can occur just after the octarepeat region (amino acid residue 90), producing the N2 and C2 fragments (Mangé et al., 2004). C2 is predominantly present in prion-infected brains (Chen et al., 1995) and has, therefore, been proposed to be susceptible to conformation change into PrP^d^ without possible subsequent inactivation by α-cleavage into C1 (Mangé et al., 2004). A third γ-cleavage occurring between amino acid residues 170 and 200 has recently been described but remains to be studied (Lewis et al., 2016). Last, PrP^c^ can be shed from the cell surface at its very C-terminal end. Even if the role of each PrP fragment is not fully understood, the cleavages appear to regulate PrP^c^ functions, notably by producing these soluble active fragments that may act as specific inhibitors (Altmeppen et al., 2012).

The emergence in the 1990s of variant Creutzfeldt–Jakob disease (v-CJD), likely caused by human dietary exposure to classical bovine spongiform encephalopathy (c-BSE) prions (Will et al., 1996), raised concerns about food safety and possible iatrogenic inter-human transmission of the disease. Secondary transmission may occur not only through medical devices, as evidenced in other human prion diseases, but also through blood products according to the peculiar presence of peripheral infectivity in v-CJD-affected patients (Ironside et al., 2000; Ritchie et al., 2004). In the United Kingdom, four probable cases of v-CJD transmission through blood transfusion confirmed this secondary risk (Turner and Ludlam, 2009). In addition, prevalence studies based on the detection of PrP^v−CJD^ in appendices have estimated that one United Kingdom inhabitant out of 2,000 would be asymptomatic carriers of v-CJD (Gill et al., 2013), which is approximately 100 times more than the total number of clinical v-CJD patients to date.

We have been developing for decades a model of experimental infection of cynomolgus macaques with prion strains as a model for the human situation, including c-BSE/v-CJD prion strains (Lasmezas et al., 1996; 2005; Herzog et al., 2004; 2005). For the sake of traceability, we continue to distinguish between c-BSE- and v-CJD-macaques according to the origin of the infectious inoculum to which they were exposed, even if they develop a similar disease. In order to assess the transfusional risk linked to v-CJD, we exposed cynomolgus macaques (n = 19) to different prion-infected blood components. Some of them (n = 5, 25%) developed c-BSE/v-CJD, but twice as many animals (n = 9, around 50% of exposed animals) developed an original, as yet undescribed, fatal neurological disease different from the classical v-CJD pattern, which we named myelopathy (Comoy et al., 2017). Their clinical expression and lesions were centered on the spinal cord, with limited brain involvement, combining bilateral necrotic lesions of anterior horns in the lower cervical spinal cord and Wallerian degeneration of posterior spinal tracts all along the spinal cord. As these primates lacked specific hallmarks of prion diseases, including neuronal lesions, astrogliosis, and vacuolization, we questioned the etiology of this syndrome: all the alternative etiological hypotheses (vascular, metabolic, nutritional, autoimmune, and infectious) that we investigated turned to be infirmed (Comoy et al., 2017). Despite the absence of detectable protease-resistant prion protein (PrP^res^), the major diagnostic marker of classical prion diseases, in the CNS of these myelopathic macaques, several arguments remain in favor of an implication of infectious prions in the development of this pathology, the main one being that these impairments can be retransmitted to mice with a pathognomonic accumulation of abnormal prion protein (Comoy et al., 2017). We, therefore, investigated the characteristics of the prion protein (PrP) in the CNS of these macaques displaying this myelopathic syndrome in comparison with PrP^c−BSE/v−CJD^ in the CNS of c-BSE/v-CJD-infected macaques and PrP^c^ in the CNS of healthy non-inoculated controls.

## Materials and methods

Ethics statement: Primates were housed and handled in accordance with the European Directive 2010/63 on the protection and welfare of animals in research and were subject to constant internal monitoring by veterinarians. Social enrichment was a constant priority, with individual activities and feeding controlled for the risk of infection. Animals were handled under anesthesia to limit stress, and euthanasia was performed for ethical reasons when animals lost autonomy. CETEA approved the present experiments (approvals 17–091 and 17–093). Due to the specificity of the macaque model, each case must be considered as a unique clinical case.

Animals and *in vivo* experiments: Captive-bred 2.5-year-old male cynomolgus macaques (*Macaca fascicularis*) were provided by Noveprim (Mauritius), checked for the absence of common primate pathogens before importation, housed in level-3 facilities (agreement numbers A 92–032–02 for animal care facilities and 92–189 for animal experimentation), and handled in accordance with national guidelines. They were all Met/Met homozygous at codon 129 of the prion protein gene (*PRNP)*. The macaques were exposed to different blood (-derived) samples through intravenous or intracerebral routes, following detailed protocols that have been previously described (Comoy et al., 2017). The animals were daily observed, and euthanasia was performed at the clinical stage when the animals lost their autonomy. The animals that developed myelopathy (n = 13) were compared to other animals that developed c-BSE (n = 18), L-BSE (n = 5), v-CJD (n = 13), or sporadic CJD (n = 3) (Herzog et al., 2004; 2005; Comoy et al., 2008; 2015) and healthy control animals (n = 7). Samples for biochemical and histological analyses were coded (two different codes), and the respective investigators were blinded during analyses. Brain and cervical spinal cord were collected and stored at −80°C, and fragments were homogenized in 5% glucose solution to obtain 20% (w/v) homogenates using the Bio-Rad TeSeE Precess 48.

Biochemical PrP^res^ analysis: Samples of the frontal cortex, occipital cortex, cerebellum, medulla, and spinal cord were serially 10-fold diluted in corresponding healthy homogenates, and PrP^res^ was purified and detected using the TeSeE ELISA kit (Bio-Rad). Theoretical optical densities (O.Ds.) were calculated by multiplying the non-saturating O.D. by the corresponding dilution factor (for example, a sample diluted 30-fold and exhibiting an O.D. = 1.200 was assigned a theoretical O.D. of 36). Occipital cortex and spinal cord samples were also tested with the Herdchek c-BSE-Scrapie Ag test (Idexx) according to the manufacturer’s instructions.

Biochemical PrP^c^ analyses: Amounts of PrP^c^ in CNS samples were evaluated using the TeSeE ELISA kit in the absence of purification. Briefly, 20% CNS homogenate was directly 10-fold diluted in the denaturation buffer C1 (which contains detergents and chaotropic agents) from the TeSeE purification kit and then heated or not for 5 min at 100°C. The resulting samples were diluted six-fold in the R6 dilution buffer of the TeSeE detection kit according to the manufacturer’s recommendations, and PrP^c^ was detected using the TeSeE detection kit.

Deglycosylation of CNS samples with PNGase F (New England Biolabs) was performed according to the manufacturer’s instructions, and revelation was performed through Western blotting with different monoclonal anti-PrP antibodies targeting distinct epitopes of the prion protein (Table 1). The same amounts of brain homogenates were loaded for each sample on the gels and were confirmed by testing the housekeeping protein actin.

**TABLE 1:** Anti-PrP antibodies used in the study.

Anti-PrP antibody	PrP epitope	Supplier
SAF32	59–89	Bertin Pharma, Montigny le Bretonneux, France
3F4	109–112	Senetek, St Louis, USA
BAR233	141–151	Bertin Pharma, Montigny le Bretonneux, France
SAF60	157–161	Bertin Pharma, Montigny le Bretonneux, France

## Results

### Myelopathic primates are devoid of classical PrP^v−CJD^

**FIGURE 1:**
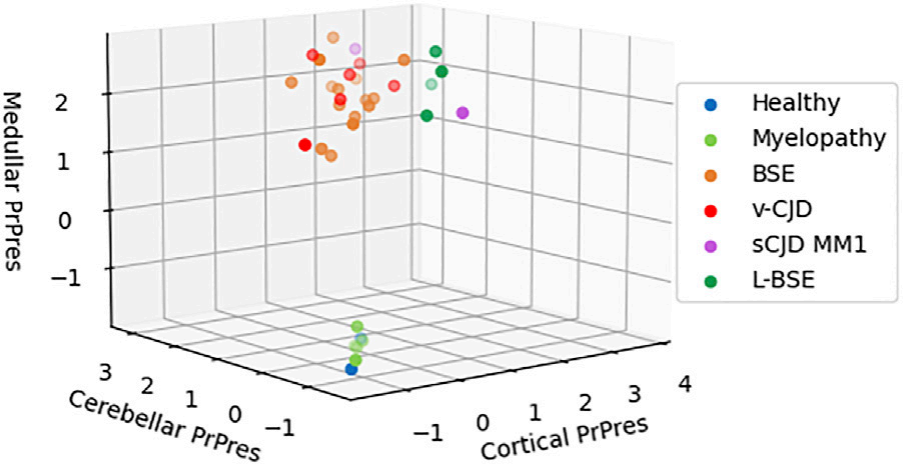
Biochemical detection of PrP^res^ in brain samples of experimentally exposed macaques. Frontal, occipital, cerebellar, and medullar samples of the brains from macaques developing the myelopathic syndrome (n = 13, dull green), c-BSE (n = 18, orange), v-CJD (n = 13, red), L-BSE (n = 5, dark green), and MM1 s-CJD (n = 3, purple) or healthy primates (n = 2, blue) were purified using the TeSeE purification kit after serial 10-fold dilutions in healthy brain. After detection, a theoretical optical density of absorbance (450 nm) was calculated by multiplying the O.D. (in the linear part of reading) by the dilution. Results are presented in a 3D plot with theoretical O.D. under a logarithmic scale. Limit of detection is an O.D. = −1 (i.e., O.D. = 0.100).

**FIGURE 2:**
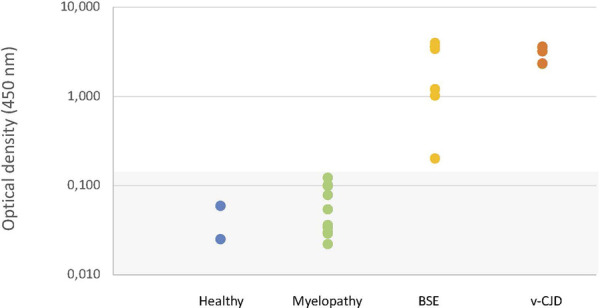
Biochemical detection of PrP^res^ in spinal cord samples of experimentally exposed macaques. Spinal cord samples from representative macaques developing the myelopathic syndrome (n = 10, dull green), c-BSE (n = 7, orange), and v-CJD (n = 5, red) or healthy primates (n = 2, blue) were tested for the presence of PrP^res^ using the TeSeE kit under the manufacturer’s instructions. Results are presented under a logarithmic scale. The gray zone determines the O.D. below the limit of detection.

**FIGURE 3:**
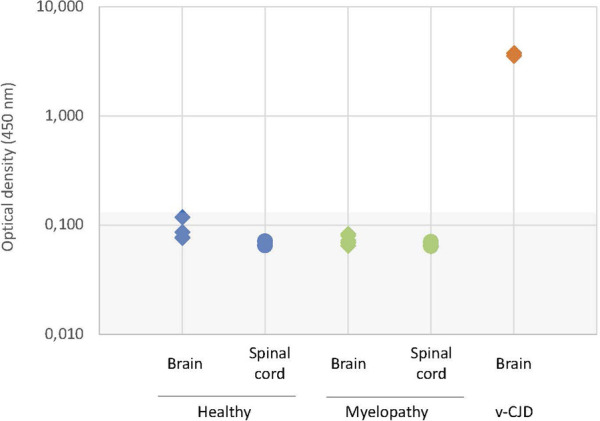
Biochemical detection of PrP^d^ in CNS samples of experimentally exposed macaques. Samples of the occipital cortex and spinal cord from representative macaques developing the myelopathic syndrome (n = 6, dull green) and v-CJD (n = 2, red) or healthy primates (n = 4, blue) were tested for the presence of PrP^d^ using the Herdchek c-BSE-Scrapie Ag test (Idexx) according to the manufacturer’s instructions. Results are presented under a logarithmic scale. The gray zone determines the O.D. below the limit of detection.

The macaques that developed the original yet undescribed myelopathic syndrome in our facilities were initially exposed to blood products issued from donors infected with c-BSE or v-CJD (Comoy et al., 2017); we, thus, first used classical biochemical approaches to assess the presence of abnormal PrP^BSE/v−CJD^ in different parts of their CNS. Our first approach was to use the diagnostic test we had previously developed for the detection of c-BSE in cattle (Deslys et al., 2001) and which was used in the whole of Europe for systematic screening of cattle at slaughterhouses during the BSE crisis. This test, the TeSeE Bio-Rad test, is based on a preliminary step of purification using proteinase K to evidence protease-resistant PrP^res^, followed by a two-step sandwich ELISA using two anti-PrP monoclonal antibodies, and it cross-reacts with primate PrP (Lescoutra-Etchegaray et al., 2015). At the brain level, no significant amount of PrP^res^ was evidenced in the (frontal and occipital) cortices, cerebellum, and medulla of myelopathic primates (all measurements were below the O.D. detection limit = 0.120), as for healthy controls, whereas high amounts were detected in the samples from other macaques experimentally infected with different strains of prions (Figure 1). It is worth noting that the topographical distribution patterns between the different areas varied depending on the prion strain, as illustrated by the groups of animals within this graph (rotating 3D plot in Supplementary Figure S1; corresponding 2D plots in Supplementary Figure S2). PrP^res^ was neither detected throughout the spinal cord of myelopathic primates but was detected in the spinal cords of c-BSE- and v-CJD-infected primates (Figure 2). The Idexx ELISA technique, which does not involve a proteolysis step and corresponds to the other diagnostic test used to detect c-BSE in cattle on the field, provided similar results: no PrP^d^ was detected in (spinal cord and occipital cortex) samples from myelopathic primates either (Figure 3), but it was present in the samples of c-BSE- and v-CJD-infected counterparts.

### Impaired expression of PrP^c^ in myelopathic primates

**FIGURE 4:**
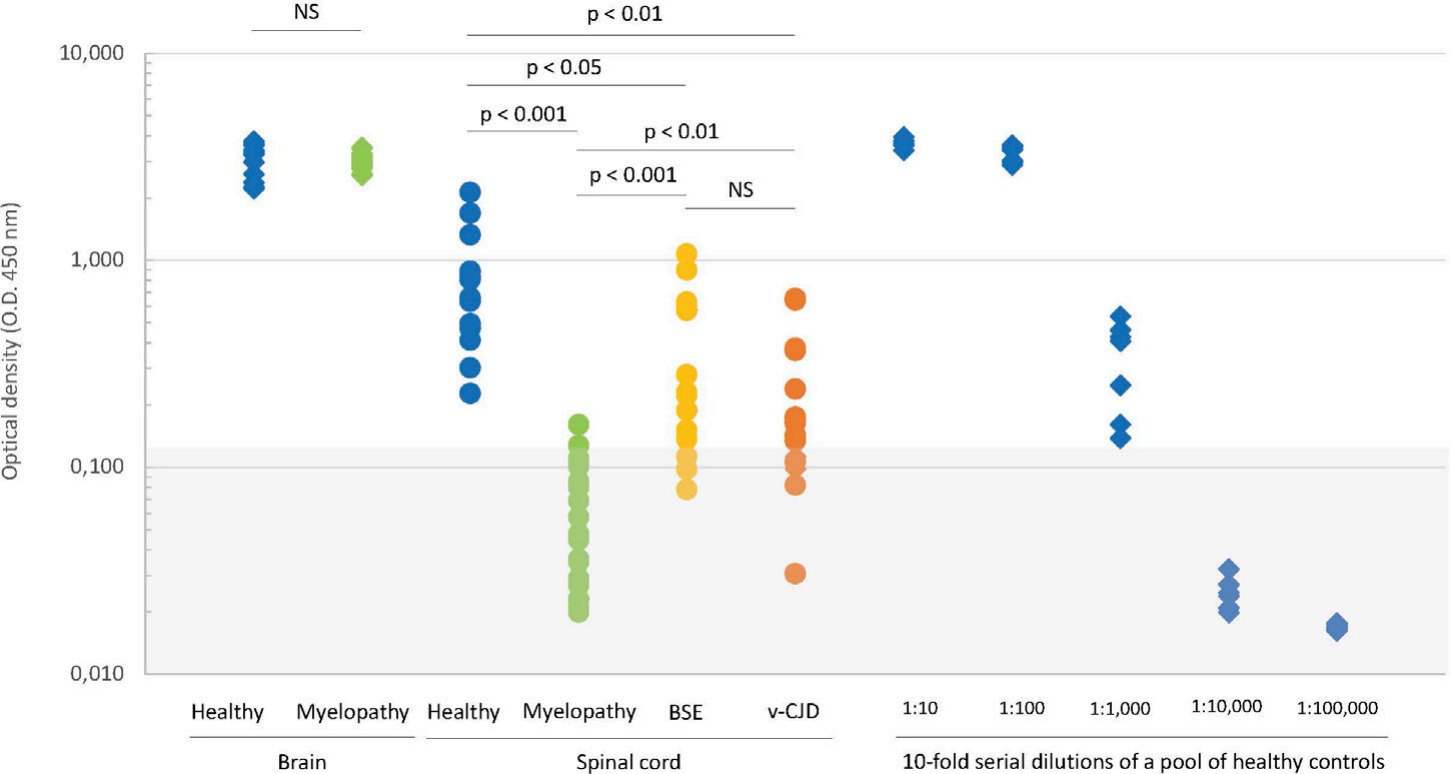
Biochemical detection of total PrP in CNS samples of experimentally exposed macaques. The amounts of PrP^c^ in samples of the brain (diamonds) and spinal cord (circles) from macaques developing the myelopathic syndrome (24 cortex samples and 19 spinal cord samples issued from 13 macaques, dull green), c-BSE (12 samples issued from 12 macaques, orange), and v-CJD (33 samples issued from 14 macaques, red) or healthy primates (19 cortex samples and 13 spinal cord samples issued from 7 macaques, blue) were estimated on crude homogenate using the TeSeE kit under the manufacturer’s instructions but without the preliminary step of purification. The six healthy brain samples exhibiting the higher O.D. were serially 10-fold diluted to serve as a reference curve for estimation of the relative amounts of PrP^c^. The gray zone determines the O.D. below the limit of detection. Significance of Student’s t-test is mentioned between groups.

We then compared the amounts of total PrP in CNS samples of the myelopathic primates to the amounts in healthy, c-BSE, and v-CJD primates by directly testing homogenates by ELISA without a prior step of proteolysis purification but after simple addition of the denaturation buffer (chaotropic agents and detergents), without the successive heating treatment that unfolds abnormal PrP. In the brain, the amounts of PrP were not statistically different between healthy and myelopathic primates (Figure 4). In the spinal cord of healthy primates, the apparent amount of total PrP was 100 to 1,000 times lower than that in their brain; in the spinal cord of myelopathic primates, the apparent amount of total PrP was below the limit of detection (O.D. < 0.120) for all but one, i.e., more than 10-fold lower than in their healthy counterparts (*p* < 0.001). In the spinal cord of c-BSE and v-CJD primates, the apparent amount of total PrP was heterogeneous, intermediate between healthy and myelopathic primates, and significantly different from these two groups, with some of them also under the limit of detection (3/18 c-BSE and 5/15 v-CJD primates). The apparent amounts of total PrP in c-BSE- and v-CJD-infected macaques were not statistically different.

**FIGURE 5:**
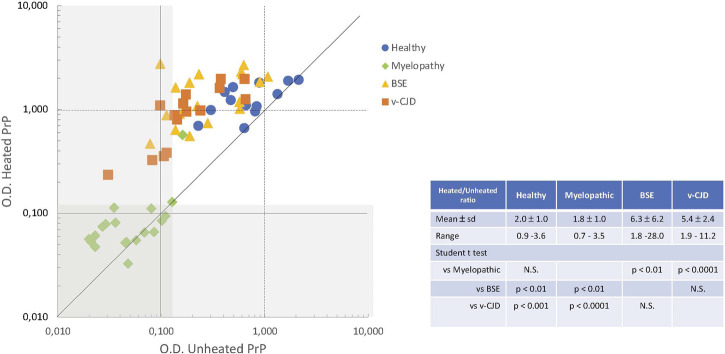
Effect of denaturation on the biochemical detection of total PrP in CNS samples of experimentally exposed macaques. Crude homogenates of the spinal cord from macaques were mixed with denaturation buffer (C1, TeSeE kit, Bio-Rad) and then heated or not for 5 min at 100°C. The amounts of PrP^c^ in the resulting samples from macaques developing the myelopathic syndrome (19 samples issued from 13 macaques, dull green diamonds), c-BSE (18 samples issued from 9 macaques, orange triangles), and v-CJD (15 samples issued from 5 macaques, red squares) or healthy primates (13 samples issued from 7 macaques, blue circles) were estimated using the TeSeE kit according to the manufacturer’s instructions. The gray zone determines the O.D. below the limit of detection.

We then investigated the significance of the apparent absence of PrP in myelopathic, c-BSE, and v-CJD primates. The peculiar folding of abnormal PrP may compromise the accessibility of the epitopes for recognition by antibodies: the unfolding of abnormal PrP is ensured in the TeSeE Bio-Rad test by the denaturation step of the purification protocol through the combined action of heating in the presence of chaotropic agents and detergents. We, therefore, tested the same samples after heating them in the presence of the denaturation buffer (Figure 5). A heated/unheated (H/U) ratio was calculated to estimate the influence of unfolding by dividing the O.D. obtained after heating by the O.D. obtained without heating. The heating step significantly increased the detection of PrP in the spinal cord samples from c-BSE- (H/U ratio = 6.3 ± 6.2; range 1.8–28.0) and v-CJD-infected animals (H/U ratio = 5.4 ± 2.4; range 1.9–11.2), in comparison to healthy (H/U ratio = 2.0 ± 1.0; range 0.9–3.6) and myelopathic (H/U ratio = 1.8 ± 1.0; range 0.7–3.5) primates, for which the effect of heating was less marked. The reversibility of PrP detection in c-BSE and v-CJD primates induced by a denaturation step argues for an impairment of the epitope detection due to the folding state of PrP^BSE/v−CJD^, whereas in myelopathic primates, this phenomenon would be related to an actual absence of detectable PrP by this technique.

**FIGURE 6:**
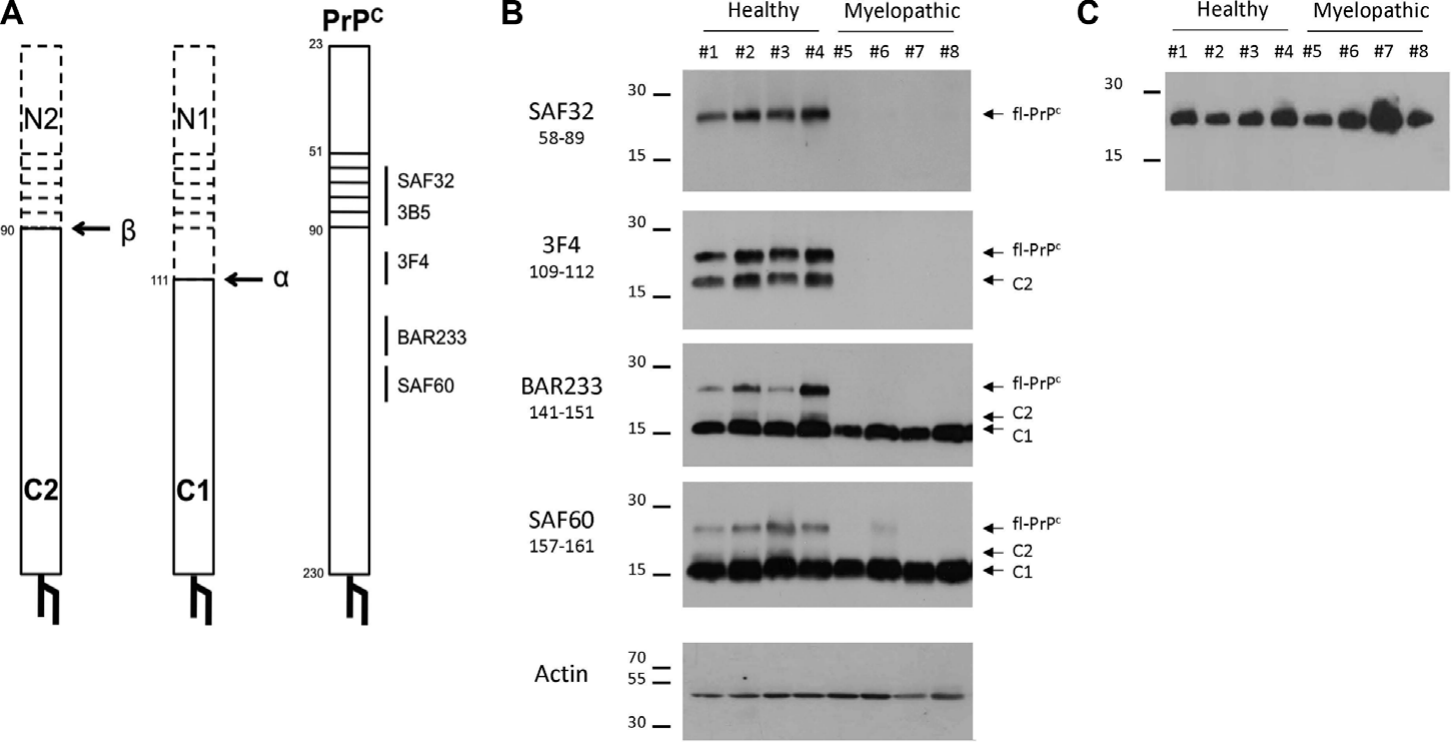
Detection of full-length PrP (fl-PrP) and derived fragments in the cervical spinal cord of primates with different anti-PrP antibodies. Binding sites of anti-PrP antibodies are shown on the schematic representations of fl-PrP^C^, C1, and C2 **(A)**. The horizontal bars represent the octarepeat region of PrP^C^. Aliquots of 10% (w/v) cervical spinal cord **(B)** or brain **(C)** homogenates were treated according to the manufacturer’s guidelines with 50 U of recombinant PNGase F and analyzed by immunoblotting with the anti-PrP monoclonal antibodies SAF32, 3F4, BAR233, and SAF60. Actin was used as a control for protein loading. Molecular weights are stated on the left (in kDa). The presented PrP profiles are from four healthy macaques (#1 to #4) and four myelopathic macaques (#5 to #8), respectively, representative of eight healthy and nine myelopathic macaques.

The detection of PrP by using the ELISA TeSeE Bio-Rad Bovine kit is based on a capture antibody recognizing the octapeptide region and a detection antibody recognizing a C-terminal part located after the hydrophobic core; the absence of detection in myelopathic primates finally shows a deficit of full-length PrP (fl-PrP). We, therefore, studied the expression of PrP fragments after deglycosylation. After deglycosylation with PNGase F (New England Biolabs) according to the manufacturer’s instructions, revelation was performed by Western blotting with different monoclonal antibodies (Table 1) that recognize different PrP fragments (Figure 6A). Three different bands of deglycosylated PrP were detected in healthy cervical spinal cords. A high-molecular-weight band around 27 kDa corresponded to full-length deglycosylated PrP^c^ (fl-PrP^c^), a lower one around 18 kDa was detected by SAF60 and BAR233 but undetected by the N-terminal antibodies SAF32 and 3F4 matched the C1 fragment, and a light band at 20 kDa was only detected in SAF60 and 3F4 fitted the C2 fragment of the PrP^c^ (Figure 6B) (Mangé et al., 2004).

In the cervical spinal cord, the amount of C1 fragment of PrP detected with SAF60 or BAR233 was similar between the populations of the myelopathic macaques and the healthy controls (Figure 6B). Conversely, the fl-PrP was undetectable or in low amounts in the cervical spinal cord of all myelopathic macaques in comparison to the healthy controls (Figure 6B), while it was present in similar amounts in the brains of both macaque populations (Figure 6C). C2 was also almost undetectable in the cervical spinal cords of myelopathic macaques compared to controls (Figure 6C).

## Discussion

In the context of our transfusion experiments in cynomolgus macaque to model the risk of iatrogenic transmission of v-CJD, we have observed an original, previously undescribed pathological entity in which no PrP^d^ accumulation was detected with the different techniques classically used to date, including not only the classical biochemical tests used for prion surveillance but also improved IHC techniques and misfolded protein amplification techniques (Comoy et al., 2017; 2018). This situation was reminiscent of our seminal observations of primary transmission of BSE from cattle to mice (Lasmezas et al., 1997), in which 50% of the animals developed a transmissible fatal disease in the absence of any detectable PrP^res^, which appeared after subsequent transmissions.

In this study, an extensive analysis of the CNS of myelopathic primates confirmed the absence of abnormal PrP^v−CJD^ in its classical, abnormally folded, protease-resistant, hydrophobic, and aggregated configuration. This study incidentally showed that the topographical pattern of PrP^res^ distribution in the CNS of macaques was not statistically different after they were infected with samples initially contaminated with c-BSE or v-CJD. This supports the idea that c-BSE and v-CJD are caused by a single prion strain (Bruce et al., 1997).

Despite the absence of classical PrP^v−CJD^, we kept on testing the prion hypothesis for this myelopathic syndrome based on its peculiar epidemiology, i.e., the occurrence of this disease only in animals exposed to blood products derived from c-BSE/v-CJD-infected donors. In both myelopathic and c-BSE/v-CJD-infected primates, the spinal cord showed reduced amounts of total PrP detectable by direct analysis in comparison to healthy controls. This observation confirms the involvement of the prion protein in the pathophysiology of the myelopathy syndrome observed. It should be noted that a decrease in PrP^c^ has also been observed in a transgenic mouse model (Dupuis et al., 2002) and human cases of amyotrophic lateral sclerosis (Kovacs et al., 2002) and is also evoked in multiple sclerosis (Scalabrino, 2022), suggesting that these two other spinal cord diseases might probably share common neurodegenerative mechanisms and the decrease in PrP^c^, involved in several neurodegenerative disorders, could be a key element.

Further analyses based on the comparison of folded and unfolded states by heat treatment in the presence of denaturing agents showed that the lack of detection in the two groups of our animals had different origins. In myelopathic primates, the lack of detection was related to a strong deficiency of full-length PrP, whereas in BSE/v-CJD primates, full-length PrP was detectable after unfolding, indicating that it was preserved from degradation by an aggregated form of PrP^d^ that impairs recognition of certain epitopes by antibodies without a prior denaturation step as classically reported (Safar et al., 2000). In both cases, the functional role of fl-PrP should be altered, but in myelopathic primates, we face a unique experimental situation in which a loss of function of PrP^c^ is clearly uncorrelated with the classical additional neurotoxic effect of PrP^d^ aggregates.

The majority of normal PrP is known to be expressed as a GPI-linked cell surface glycoprotein, and surprisingly, in transgenic mice expressing PrP devoid of GPI anchoring, a classical prion strain (RML scrapie) does not induce clinical disease despite the presence of amyloid PrP^res^ and replication of scrapie infectivity (Chesebro et al., 2005). In contrast, in our experimental model without amyloid PrP^res^, we observed clinical signs linked to a spinal cord involvement that is consistent with lesions in the cervical spinal cord in which the majority of PrP has lost its N-terminus, suggesting a link with a loss of function of this corrupted PrP.

Several studies report that the truncation of its N-terminus somehow alters the normal biological activity of PrP^c^ in a way that produces neurotoxic effects (Shmerling et al., 1998; Li et al., 2007; Sonati et al., 2013; Le et al., 2019). However, all these artificial transgenic models question their relevance to a natural situation that integrates multiple highly regulated molecular mechanisms (Wulf et al., 2017). Neuronal expression of membrane-anchored PrP^c^ is known to be required for prion-induced neurodegeneration *in vivo* (Brandner et al., 1996; Mallucci et al., 2003; Chesebro et al., 2005). The elegant seminal experiments of grafting neural tissue overexpressing PrP^C^ into the brain of PrP-deficient mice (*Prnp0*/*0*) demonstrated not only that after prion infection (scrapie RML strain), only the grafts were destroyed with PrP^sc^ toxic accumulation but also that surrounding tissue devoid of PrP^C^ remained healthy despite the diffusion of PrP^sc^ aggregates from the grafts (Brandner et al., 1996). Here, we face a reverse situation in myelopathic primates, with the absence of toxic aggregated PrP and a pathology associated with the natural N-terminal truncation of the majority of PrP^c^ in their spinal cord. It should be noted that the absence of N-terminal regions of PrP^c^ is suspected to induce vulnerability to the ischemic brain, heart, or kidney damage, with higher apoptotic cell death and higher oxidative stress in the damaged tissues (Hara and Sakaguchi, 2020)*.* The preferential involvement of the cervical spinal cord in the lesions of our myelopathic primates might be related to a higher vulnerability of this portion to trauma, according to its daily solicitation.

From an evolutionary point of view, the remarkable conservation of PrP^c^ in vertebrates, especially in mammals, its high turnover rate, and its expression in all tissues strongly suggest important physiological functions of this protein (van Rheede et al., 2003; Aguzzi et al., 2008; Gasperini and Legname, 2014; Hirsch et al., 2017). Among the multiple functions suggested for PrP, the maintenance of myelin in peripheral nerves is the main role gathering relative consensus. Mice ablated for PrP (first-generation PrP knockout mice and prnp-ablated mice generated through genome editing) (Bremer et al., 2010; Nuvolone et al., 2016) and goats lacking PrP (Skedsmo et al., 2020) develop a progressive peripheral demyelinating neuropathy, whose mechanism is now foreseen. The N-terminal flexible tail of PrP activates the adhesion of G-protein-coupled receptor Adgrgr6 on Schwann cells, whose activation is necessary for the maintenance of myelin (Küffer et al., 2016).

In contrast to this well-documented situation in the peripheral nerves, the role of PrP in myelin physiology at the level of the central nervous system is less clear in the literature. Oligodendrocytes are classically considered resistant to infection, and neuropathological alterations, although extensive and variable, have been considered limited to cortical and subcortical gray matter in human CJD. However, cases of the “panencephalopathic” type of CJD initially reported in Japan (Mizutani et al., 1981) suggested the possibility of primary involvement of white matter, at least with some prion strains, and the description of oligodendrocytes engulfed within the cytoplasm of hypertrophic astrocytes (emperipolesis) was later reported to be common in the cerebral white matter of sporadic CJD patients (Shintaku and Yutani, 2004). More recently, oligodendrocyte vulnerability and myelin alterations were reported in the advanced stages of murine CJD (Andrés-Benito et al., 2021). Our data suggest that PrP^c^ would also play a key role in the maintenance of myelin in the central nervous system, and its loss of function would be involved in the myelopathic lesions observed in the spinal cord with demyelination of the posterior tracts, which is coherent with the loss of sensitivity that we observed in our primates.

To our knowledge, here we observe the first experimental situation of detection of a specific biochemical signature in a prion disease without detectable PrP^res^. However, while the natural resistance to degradation of aggregated PrP^d^ allows detection of even minute amounts among normal cellular PrP, here we are in the opposite situation of an abnormally fragile corrupted PrP that becomes undetectable among total cellular PrP unless it becomes the majority. This is the case in the spinal cord lesions that we tested.

All the prion disease entities that have been described so far for decades are based on PrP^c^ that undergoes conformational changes towards more structured states, which provide to the resulting PrP^d^ different properties that tend to overall increase their resistance to degradation compared to PrP^c^; even diseases that are described as intermediate or protease-sensitive forms (Nor98 and vPSPr) are associated with PrP^d^ that exhibit a more structured conformation than PrP^c^. These properties pave the way for different approaches used for decades to diagnose prion diseases in the absence of conformational antibodies. The less-resistant physiological PrP is classically eliminated so that PrP^d^ remains the only form detectable by specific anti-PrP antibodies.

Here, the PrP in the spinal cord of the myelopathic primates is apparently cleaved in greater amounts than the classical physiological PrP^c^ in the spinal cord of their healthy counterparts, indicating a greater sensitivity of this abnormal PrP to proteolytic processes. We hypothesized that in these animals, pathological conformational changes would occur that lead to pathological PrP with less structured conformations than physiological PrP^c^, which would then make this PrP^d^ more susceptible to enzymatic degradation and, thus, to natural elimination.

Furthermore, this excess of C1 production might be considered a cellular mechanism of defense against prion infection. Our studies of physiological PrP fragments have shown that depletion of fl-PrP in the spinal cord of our myelopathic primates is associated with a depletion of the C2 fragment, while C1 apparently remains present in normal amounts; this abnormal pattern suggests an imbalance of α- and β-cleavages compared to healthy macaques, as the signature of a profound alteration in PrP physiology in contrast to the normal pattern of fl-PrP expression in the brains of these animals.

Although several studies report conflicting results regarding the roles of ADAM9, ADAM10, and ADAM17 in α-cleavage of PrP^c^, it is generally admitted that alpha-cleavage and C1 are protective against prion misfolding (Westergard et al., 2011). Furthermore, the α-cleavage site located in the neurotoxic domain is suspected to be involved in the misfolding of PrP^c^ into PrP^d^; therefore, the absence of this domain in C1 has been proposed to explain why only fl-PrP and C2 could be misfolded into PrP^d^ (Chen et al., 1995). According to this hypothesis, in our myelopathic macaques, the disappearance of fl-PrP and the absence of C2 would protect against any mechanism of prion conversion to PrP^d^ but would also result in a loss of the physiological signal for the maintenance of myelin. This molecular shift could then correspond to a natural cellular mechanism for fighting a prion infection, with three possible outcomes:

- In most situations, this mechanism would prevent low prion infectious doses from initiating a perennial replication and allow the elimination of residual infectivity by cellular catabolism in individuals who would remain apparently healthy
- At the other extreme, if this mechanism is saturated by a high infectious dose and/or an aggressive prion strain, the infection would lead to the onset of classical prion disease
- Finally, in the third scenario, with low infectious doses and a sufficiently aggressive strain, such as the one responsible for vCJD, this mechanism is sufficient to contain the infection but not to eliminate it, and such low-level replication keeps this physiological mechanism abnormally active in the long term with the pathological consequences on the myelin that we observe in our myelopathic animals.

In conclusion, we have identified here for the first time a new specific biochemical signature that supports the prion origin of the myelopathic syndrome that we observe in our macaques exposed to contaminated blood products and potentially a natural cellular defense mechanism against prions that failed in diseased animals. This myelopathic syndrome opens new perspectives in the field of prion diseases towards alternative conformations of abnormal PrP never studied before, i.e., abnormal forms less resistant than the physiological PrP and, therefore, complex to evidence. This concept justifies the need to revisit several neurodegenerative diseases, in particular those affecting the spinal cord, in light of our observations in primates, for which the search for abnormal PrP by conventional techniques is inadequate.

## Figures and Tables

**Table:** 

**c-BSE**	Classical Bovine spongiform encephalopathy
**CNS**	Central nervous system
**CWD**	Chronic wasting disease
**fl-PrP**	Full-length PrP
**GPI**	Glycophosphatidylinositol
**PrP**	Prion protein
**PrPc**	Physiological, cellular prion protein
**PrPd**	Pathological prion protein associated to a prion disease
**PrPres**	Abnormal PrP resistant to proteolysis
**PrPv-CJD**	Pathological prion protein in individuals affected by the variant of Creutzfeldt–Jakob disease
**v-CJD**	Variant Creutzfeldt–Jakob disease

## Data Availability

The raw data supporting the conclusion of this article will be made available by the authors, without undue reservation.

## Supplementary material

SUPPLEMENTARY VIDEO S1 Biochemical PrP^res^ detection in brain samples of experimentally exposed macaques. Frontal, occipital, cerebellar, and medullar samples of the brains from macaques developing the myelopathic syndrome (n=13, dull green), c-BSE (n=18, orange), v-CJD (n=13, red), L-BSE (n=5, dark green), MM1 s-CJD (n=3, purple), or healthy primates (n=2, blue) were purified using the TeSeE purification kit after serial 10-fold dilutions in healthy brain. After detection, a theoretical optical density of absorbance (450 nm) was calculated by multiplying the O.D. (in the linear part of reading) by the dilution. Results are presented in a moving 3D plot with theoretical O.D. under a logarithmic scale. Limit of detection is an O.D. = -1 (i.e., O.D. = 0,100).

SUPPLEMENTARY FIGURE S1 Biochemical PrP^res^ detection in brain samples of experimentally exposed macaques. Frontal, occipital, cerebellar, and medullar samples of the brains from macaques developing the myelopathic syndrome (n=13, dull green), c-BSE (n=18, orange), v-CJD (n=13, red), L-BSE (n=5, dark green), MM1 s-CJD (n=3, purple), or healthy primates (n=2, blue) were purified using the TeSeE purification kit after serial 10-fold dilutions in healthy brain. After detection, a theoretical optical density of absorbance (450 nm) was calculated by multiplying the O.D. (in the linear part of reading) by the dilution. Results are presented through different 2D plots, each associating different pairs of samples, with theoretical O.D. under a logarithmic scale. Limit of detection is an O.D. = -1 (i.e., O.D. = 0,100).
