# Supplementary figures and images for: Unexpected decrease of full-length prion protein in macaques inoculated with prion-contaminated blood products
Source: Front Mol Biosci. 2023 May 5;10:1164779. doi: 10.3389/fmolb.2023.1164779 (PMC10196267; doi:10.3389/fmolb.2023.1164779)

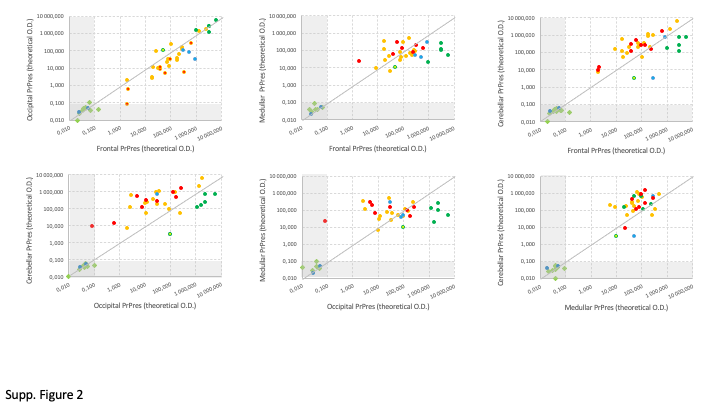

Supplement: Supplementary file 1 [file Image1.TIFF]
